# Low-dose interleukin-2 can improve salivary secretion but not lymphocyte infiltration of salivary glands in a murine model of Sjögren’s syndrome

**DOI:** 10.1186/s12865-022-00524-1

**Published:** 2022-10-16

**Authors:** Junsong Wen, Fenglin Zhu, Xi Yu, Hualing Xie, Chengyin Li

**Affiliations:** 1 Department of Rheumatology, Chongqing Hospital of Traditional Chinese Medicine, Chongqing, 400021 China; 2 Hunan University of Traditional Chinese Medicine, Changsha, 410208 China; 3 Basic Research Department of Traditional Chinese Medicine and Pharmacy, Chongqing Hospital of Traditional Chinese Medicine, Chongqing, 400021 China; 4 Department of Traditional Chinese Medicine, Chongqing College of Traditional Chinese Medicine, Chongqing, 400000 China

**Keywords:** Sjögren’s syndrome, Interleukin-2, Salivary gland, Exocrine function, Immune-related damage

## Abstract

**Introduction/aim:**

Effects of low-dose interleukin-2 (IL-2) on the exocrine glandular glands of Sjögren’s syndrome are unknown. The aim of this study was to investigate the effects of low-dose IL-2 on salivary gland structure and function in a murine model of Sjögren’s syndrome.

**Materials and methods:**

Non-obese diabetic/Ltj (NOD) mice were used as the animal model of Sjögren’s syndrome, and low-dose IL-2 or phosphate buffered saline was administered subcutaneously from 5 weeks of age, while ICR mice were used as controls. Some mice were sacrificed at 9 weeks of age, while the other mice that continued to receive treatment were sacrificed at 23 weeks. We determined the salivary flow rate of mice every 3 weeks during the intervention. After the mice were sacrificed, one submandibular gland was removed for pathological evaluation, while the other submandibular gland was used to measure the levels of 25 cytokines by Luminex technology. Cervical lymph nodes and spleens were examined by flow cytometry for the proportions of CD8^+^ T cells and Treg cells.

**Results:**

The results showed that the salivary flow rate of NOD mice was slower than that of control-group mice, and there were more pathological changes in the submandibular gland. The levels of many cytokines in the submandibular gland were elevated. The proportion of CD8^+^ T cells in the cervical lymph nodes and spleens was increased; however, the proportion of Treg cells was decreased. After treatment with IL-2, the exocrine function of the salivary glands of mice was improved. IL-2 also promoted the proliferation of Treg cells in the cervical lymph nodes and spleens, but it did not alter the extent of lymphocyte infiltration in the submandibular gland. The levels of cytokines in the submandibular glands, as well as the proportion of CD8^+^ T cells in the cervical lymph nodes and spleens, were unchanged significantly after IL-2 treatment.

**Conclusion:**

Our results demonstrate that treatment with low-dose IL-2 improves the secretory function of the exocrine glands of mice with Sjögren’s syndrome, but it does not reverse the structural damage of the exocrine glands.

## Background

Sjögren’s syndrome (SS) is a chronic, systemic, and autoimmune disease [1]. Its average prevalence is 0.2% in the adult population. The affected population is mainly comprised of middle-aged women, with a male-to-female ratio of about 1:9. The symptoms of dry mouth, dry eyes, and other symptoms seriously affect the quality of life of patients. In addition, at least one third of patients may have extensive organ damage. Lymphoma is a common complication in patients with SS that seriously threatens the survival of patients [2–7]. The pathogenesis of SS is complex, and there is a lack of effective therapeutic drugs. Current treatment strategies mainly improve clinical symptoms and suppress autoimmune responses [8–11]. However, medications that improve symptoms cannot delay the progression of the disease, and the drugs that suppress the autoimmune response often fail to improve symptoms and increase the risk of infection in patients. Therefore, there is an urgent need to identify new therapeutic drugs.

In recent years, the use of low-dose interleukin-2 (IL-2) to treat autoimmune diseases has attracted widespread attention. Because of its strong immunomodulatory effect without increasing the risk of infection, IL-2 has been used in the treatment of type 1 diabetes and systemic lupus erythematosus, and its inherent mechanism is that low doses of IL-2 can promote the proliferation of Treg cells and restore the balance of immune system [12, 13]. The immunological imbalance between Treg cells and Th17 cells is at the core of the pathogenesis of SS and systemic lupus erythematosus. Some researchers have reported that low-dose IL-2 treatment can induce the proliferation of Treg cells in the peripheral blood of SS patients, thereby re-establishing the balance of immune system [14]. Treg cells have an immunosuppressive role, and theoretically, the proliferation of Treg cells can alleviate the immune damage caused by SS. However, the lack of studies of low-dose IL-2 in protecting the structure and the function of exocrine glands in SS patients leaves the question open. Moreover, IL-2 is an activator of CD8^+^ T cells, and an up-regulation of the number and the function of CD8^+^ T cells is one of the causes of SS [15, 16]. However, it is unclear whether low-dose IL-2 can aggravate the progression of SS by stimulating the activation of CD8^+^ T cells.

In this study, we treated the model mice with SS with low dose of IL-2. The aim of this study was to determine whether IL-2 could improve the structural and functional damage of the exocrine grands of mice with SS.

### Methods animals

Four-week-old non-obese diabetic/Ltj (NOD/Ltj) and ICR female mice were purchased from GemPharmatech Co., Ltd. (Nanjing, China). Because the characteristics of NOD/Ltj mice with SS are similar to those of humans with the disease, they are widely used as an animal model of SS. We used ICR mice as controls due to their similar genetic backgrounds. There were 5 mice in each group. All animal experiments were carried out in accordance with the guidelines of the Animal Protection and Use Committee and the Experimental Animal Welfare and Ethics Committee of Chongqing Hospital of Traditional Chinese Medicine.

### Treatment with low-dose recombinant murine interleukin-2

**Fig. 1:**
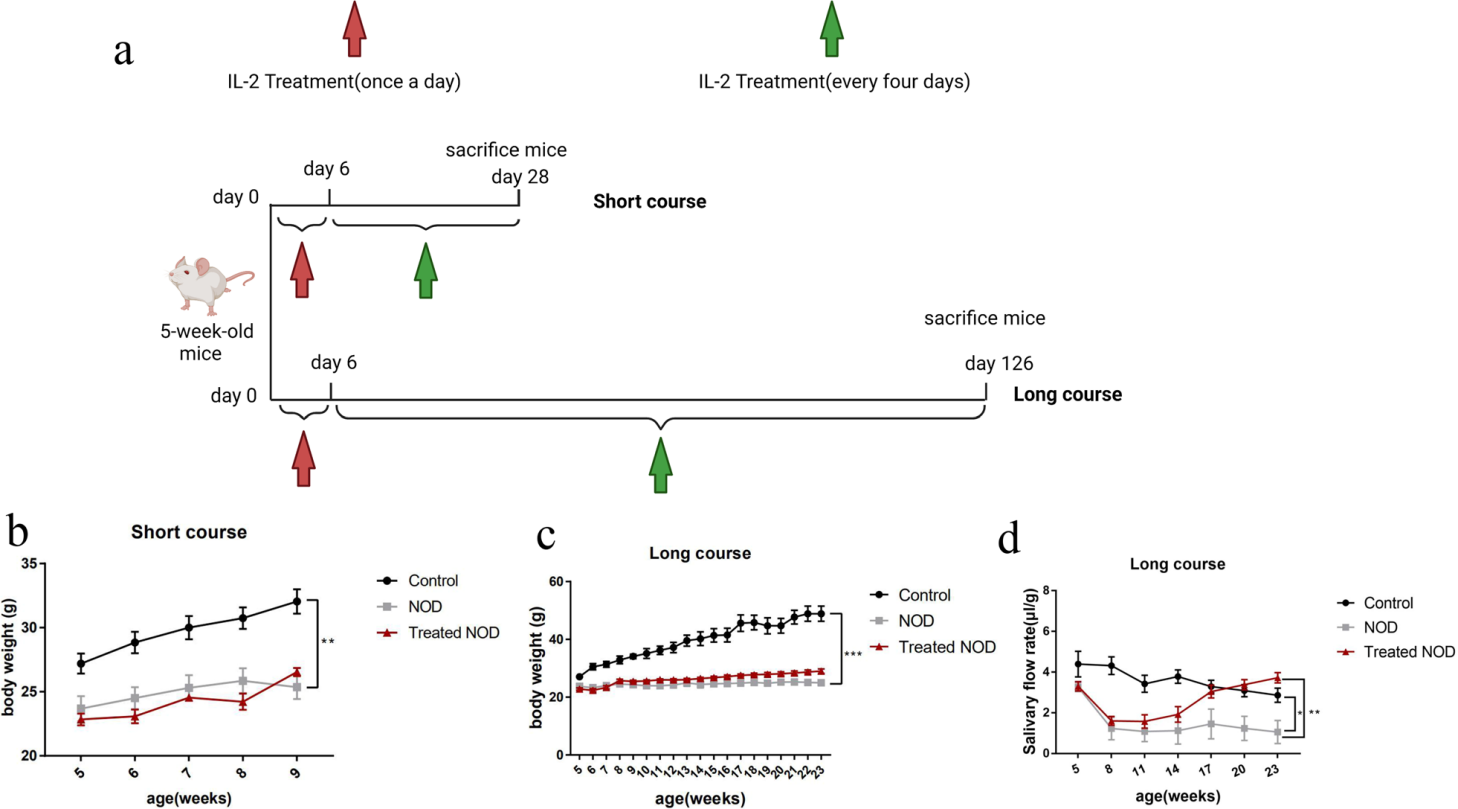
Low-dose IL-2 can improve salivary secretion in a Sjögren’s syndrome model. **a** Experimental schema of IL-2 treatment: Mice were injected subcutaneously with IL-2, once daily for the first 6 days, and every 4 days for the subsequent maintenance treatment. The mice in the short-course and long- course group group were sacrificed at the age of 9 weeks and 23 weeks respectively (n = 5). **b and c** Changes in body weight parameters of **b** short-course group and **c** long-course group(n = 5). **d** Changes in salivary flow rate per unit body weight of mice treated with IL-2 (μl/g) (n = 5)

After one week of adaptive feeding, NOD/Ltj mice were injected subcutaneously with recombinant murine interleukin-2 (rMuIL-2) at 25 ng/g body weight or phosphate buffered saline (PBS). The dosage is based on previous experiments and previous relevant literature [17]. rMuIL-2 was purchased from Prime Gene Corporation (Shanghai, China). Female ICR mice of the same age were subcutaneously injected with PBS. Injections were given once a day for the first 6 days and every 4 days thereafter. The injection protocol was terminated in mice at 9 weeks of age in the short-term treatment group and at 23 weeks of age in the long-term treatment group (Fig. 1a). The mice were sacrificed at 12 h (short-course group) or 48 h (long-course group) after the last injection of IL-2, and the tissues or organs, such as submandibular glands, spleens, and cervical lymph nodes, were removed for further analysis.

### Measurement of salivary flow rate

After anesthesia, the mice were intraperitoneally injected with pilocarpine at 2 mg/kg body weight. Saliva specimens were immediately collected. In brief, the mice were held in an inverted position, and tiny drainage tubes were placed at the bottom of their mouths, allowing the saliva to flow into the tiny centrifuge tubes. Saliva was collected continuously for 15 min, and the volume of saliva was measured with a micropipette after centrifugation. We measured the salivary flow rate of mice at 5, 8, 11, 14, 17, 20, and 23 weeks of age (μL /15 min). Finally, the saliva flow rate was calculated by µl/gram body weight.

### Histological analysis

After the mice were sacrificed, one submandibular gland was removed, immediately fixed with 4% paraformaldehyde, and embedded in paraffin. All mice were sacrificed by exsanguination under anesthesia by 5% isoflurane. All paraffin-embedded tissue blocks were cut to generate 2-μm thick sections, followed by staining with hematoxylin and eosin for 3 min. Sections were viewed and images were acquired with a microscope (BX53, Olympus, Tokyo, Japan), and the area of ​​the lymphocytic infiltration lesion in each field was calculated using ImageJ software (National Institutes of Health, Bethesda, MD, USA) (50 lymphocytes per 4 mm^2^ of tissue). Finally, the ratio of the submandibular gland lymphocyte infiltration area to the total area was calculated.

### Flow cytometry

The spleens and cervical lymph nodes of mice were removed and placed in RPMI 1640 medium for digestion. Lymph nodes were digested with 0.25 mg/mL type IV collagenase and shaken in a 37 °C incubator for 15 min. After the removal of the erythrocyte lysis buffer (Beyotime Biotechnology, Beijing, China), the splenocyte suspension was washed with PBS. The resuspended cells, as well as the spleen and cervical lymph node cell suspensions, were filtered through 40-μm cell strainers. To identify cell surface markers, the cell suspensions were incubated with Fc receptor blocker (Miltenyi Biotec) for 15 min at 4 °C. The suspensions were incubated with mouse antibodies for 30 min at 4 °C as follows: CD45 (DZ594, BioLegend, clone 30-F11), CD8 (PerCP/Cy5.5, BioLegend, clone 53-6.7), CD4 (PE, BioLegend, clone GK1.5), Foxp3 (Alexa Fluor® 647, BioLegend, clone 150D), Fixable viability dye eFluor (eBioscience) was added to the suspension to differentiate the dead cells from the live cells. To detect the levels of intracellular cytokines, the cells were pre-incubated with PMA (0.9 nM, Sigma-Aldrich, Steinheim, Germany), ionomycin (0.5 μg/ml, Sigma-Aldrich), brefeldin A (0.5 mg/ml, eBioscience, Santiago, California, USA), and monensin A (2 mM, eBioscience) in RPMI 1640 medium at 37 °C for 6 h. Subsequently, the cells were fixed and permeabilized for 30 min, followed by incubation at room temperature for 30 min. The cell suspensions were incubated with mouse antibody at 4 °C as follows: interferon-γ (APC, BioLegend, clone XMG1.2). Flow cytometry was carried out with the FACS Aria II sorter (BD Biosciences, Franklin Lake, New Jersey, USA), and data were analyzed by FlowJo_v10.6.2 software.

### Luminex immunoassays

After the submandibular glands were homogenized, the total protein concentrations of the lysate were determined, and the total protein concentrations were adjusted to 1 mg/ml. Immunoassays were performed using the 25-Plex magnetic assay (Cytokine & Chemokine 25-Plex Mouse ProcartaPlex Panel 1, eBioscience, Thermo Fisher Scientific, Waltham, MA, USA), according to the manufacturer’s instructions. The levels of 25 cytokines and chemokines, including IL-1β, IL-2, IL-5, IL-6, IL-9, IL-10, IL-12p70, IL-13, IL-17A,IL-18, IL-22, IL-23, IL-27, TNF -α, IFN-γ, CCL2, CCL3, CCL4, CCL5, CCL7, CCL11, CXCL1, CXCL2, CXCL10 and GM-GSF, were examined. The concentration of each cytokine or chemokines was expressed as pg/mL, and commercially procured control samples for each analyte were used in parallel to ensure the reliability of the results. In brief, after adding 50 μl of the diluted magnetic bead suspension to each well of the 96-well plate, the plate was affixed to a magnetic plate rack, and 150 μl of the washing buffer was used to wash the magnetic beads. Next, 50 μl of the standard solution and the sample was added to the standard wells and the sample wells, respectively, and the plate was incubated for 2 h at room temperature on a shaker at 800 rpm. This was followed by the washing of each well with 150 μl of washing buffer three times. Next, 25 μl of the diluted biotin antibody mixture was added to each well, followed by incubation at room temperature for 30 min on a shaker at 800 rpm. This was followed once again by the washing of each well with 150 μl of washing buffer three times. Next, 50 μl of streptavidin–phycoerythrin was added to each well, and the plate was incubated for 30 min at room temperature on a shaker at 800 rpm. Finally, 150 μl of the washing buffer was added to each well, and the plate was incubated for 2 min at room temperature on a shaker at 800 rpm. The plate was then inserted into the Luminex 200 instrument (Luminex Corp., Austin, TX, USA), and the plate was read within 90 min.

### Statistical analysis

GraphPad 7.00 software (California, US) was used for statistical analysis, and data were expressed as the mean ± SEM. The main results were analyzed by the independent sample t test. Significant differences were taken at *p* < 0.05.

## Results

### Treatment with a low dose of IL-2 improved the saliva flow rate

To clarify the effects of low-dose IL-2 on salivary gland function, we divided mice into the control group, the NOD group, and the Treated NOD group, and these groups were treated as indicated from 5 weeks of age. Phosphate buffered saline was subcutaneously injected, while the Treated NOD group received IL-2. At 5, 8, 11, 14, 17, 20, and 23 weeks of age, the salivary flow rate of the mice in the three groups was determined. In terms of body weight, the weight of mice in the control group increased rapidly, and the weight of mice in the control group was higher than that in the NOD group after the treatment of both the short course group and the long course group. Compared with the NOD group, there was no significant difference in body weight of IL-2 treated mice (Fig. 1b, c). The saliva flow rate results showed that the salivary flow rate was low in both NOD group and Treated NOD group, while it was high in the control group. With the passage of time, the salivary flow rate did not change significantly in control-group and NOD-group mice, although it was high in mice of the control group. The salivary flow rate of the NOD group was low, while it increased significantly in the Treated NOD group mice from the 14th week, and the salivary flow rate between the NOD group and the treatment group began to show a statistical difference (Fig. 1d).

### Treg frequency was up-regulated after low-dose IL-2 therapy

**Fig. 2:**
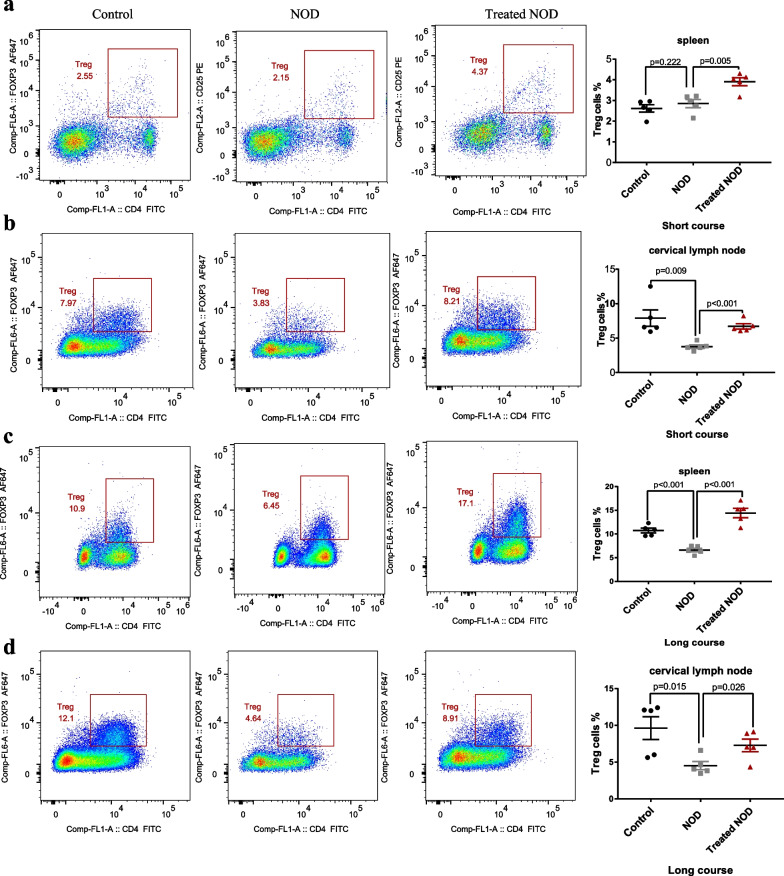
Treg proportions was up-regulated after low-dose IL-2 therapy. **a** Proportions of Treg cells in the spleen of the short-course group (n = 5). **b** Proportions of Treg cells in cervical lymph nodes of short-course group (n = 5). **c** Proportions of Treg cells in the spleen of long-course group (n = 5). **d** Proportions of Treg cells in cervical lymph nodes of long-course group (n = 5)

Regulatory T cells (Treg cells) are a group of cells involved in the regulation of autoimmunity, mainly playing a negative immunomodulatory role. The lack or reduction of the Treg cells population is associated with the pathogenesis of many autoimmune diseases [18]. IL-2 can promote the proliferation of Treg cells [14, 19]. Therefore, we further investigated the effect of IL-2 on the proportions of Treg cells in cervical lymph nodes and spleens of mice with SS. The results showed that, compared with the control group, the proportions of Treg cells in cervical lymph nodes and spleens of mice in the NOD group unchanged or decreased, while the proportions of Treg cells in cervical lymph nodes and spleens of mice in the Treated NOD group increased (Fig. 2a–d).

### ***Treatment with a low dose of IL-2 failed to reduce the proportion or function of CD8***^+^***T lymphocytes***

**Fig. 3:**
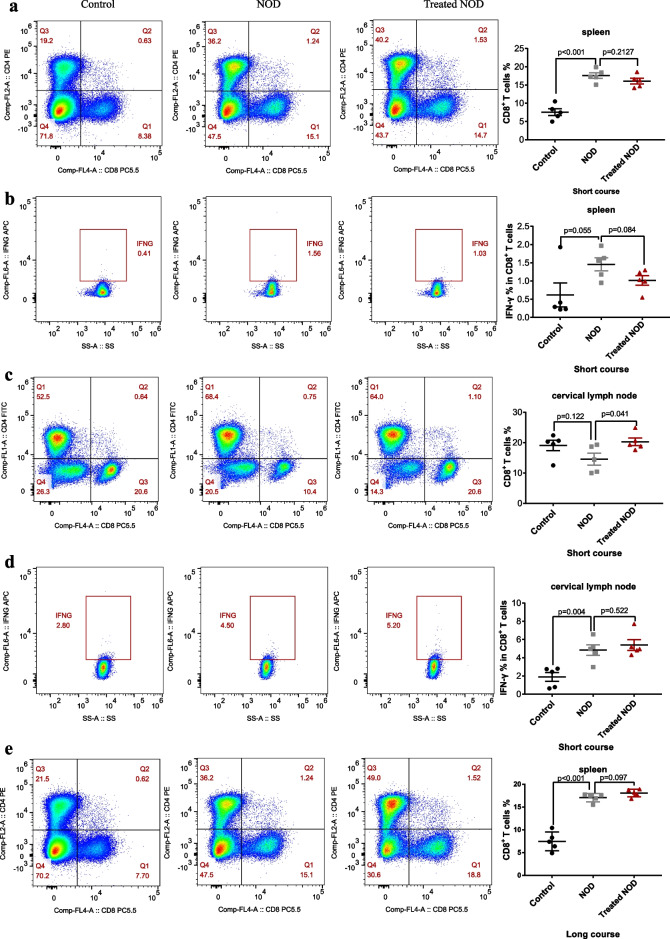

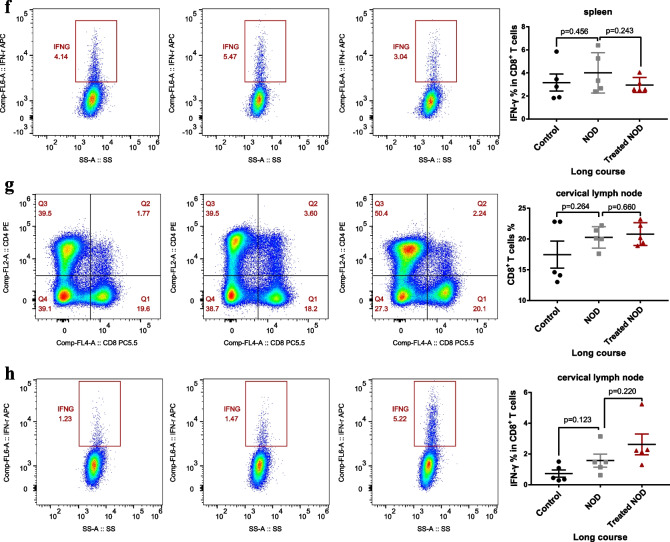
Treatment with low-dose of IL-2 failed to reduce the proportion or function of CD8^+^ T lymphocytes. The proportion of CD8^+^ T cells in the spleen of **a** the short-course group and **e** the long-course group. The proportion of CD8^+^ T cells that secrete IFN-γ in the spleen of **b** the short-course group and **f** the long-course group. The proportion of CD8^+^ T cells in the cervical lymph node of **c** the short-course group and **g** the long-course group. The proportion of CD8^+^ T cells that secrete IFN-γ in the cervical lymph node of **d** the short-course group and **h** the long-course group

CD8^+^ T lymphocytes are a complex group of cells with diverse phenotypes that have important roles in tumors development, viral infections, chronic inflammation, and autoimmunity [20–25]. Multiple studies have revealed that CD8^+^ T lymphocytes promote acinar damage in exocrine glands in patients with SS [26]. IL-2 is an activator of CD8^+^ T lymphocytes. Therefore, although the dose of IL-2 used in this study was low, it was important to determine whether CD8^+^ T lymphocytes were further activated after long-term and multi-dose administration of IL-2. Therefore, we investigated the effect of IL-2 on the proportion of CD8^+^ T lymphocytes in cervical lymph nodes and spleens of mice with SS. The results showed that, compared with the control group, the proportions of CD8^+^ T lymphocytes in the spleens of NOD mice in both the long-course and short-course groups was significantly increased, although the function of CD8^+^T cells was not increased (Fig. 3a, b, e, f). The results also showed that compared with the control group, the function of CD8^+^ T lymphocytes in the cervical lymph nodes of NOD group mice was significantly increased in the short-course group, and low doses of IL-2 did not significantly reduce the function of CD8^+^T lymphocytes, compared with the NOD group (Fig. 3d). Notably, in the short-course group, the number of CD8^+^T lymphocytes in the cervical lymph nodes after IL-2 treatment not only did not decrease but significantly increased compared with NOD group (Fig. 3c). This effect was not seen in the long-course group, though (Fig. 3g, h).

### Low-dose IL-2 failed to alleviate immunopathological damage of the submandibular gland in Sjogren’s syndrome model mice

**Fig. 4:**
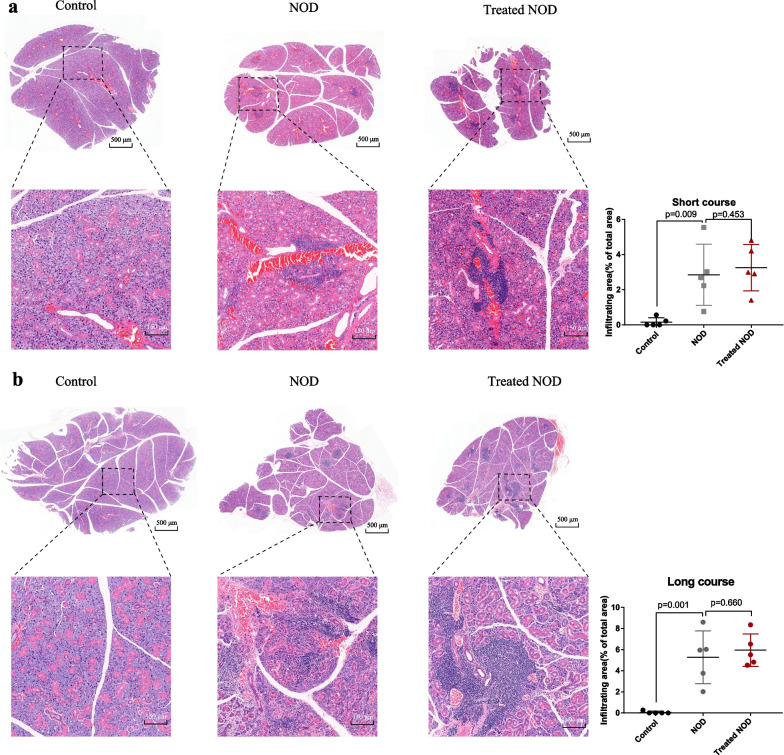
Low-dose IL-2 failed to alleviate immunopathological damage of the submandibular gland in Sjogren’s syndrome model mice. **a** The pathological map and lymphocyte infiltration rate of the submandibular gland in the short-course group (n = 5). **b** Pathological map and lymphocyte infiltration rate of submandibular gland lymphocyte infiltration in the long-course group (n = 5)

The above results show that IL-2 can promote the proliferation of Treg cells in SS model mice, but it has no inhibitory effect on the increase of CD8^+^ T lymphocytes. This made us even more curious about whether low doses of IL-2 could protect the submandibular gland from immunopathological damage. Therefore, we analyzed the effects of short-course and long-course low-dose IL-2 on submandibular gland pathology in SS model mice. The results showed that the infiltration ratio of submandibular gland lymphocytes in NOD group mice was much larger than that in the control group mice, and the infiltration ratio of submandibular gland lymphocytes in Treated NOD mice was not significantly different from that in the NOD group (Fig. 4a, b).

### Low dose IL-2 failed to affect the levels of multiple cytokines or chemokines of the submandibular gland in SS model mice

**Fig. 5:**
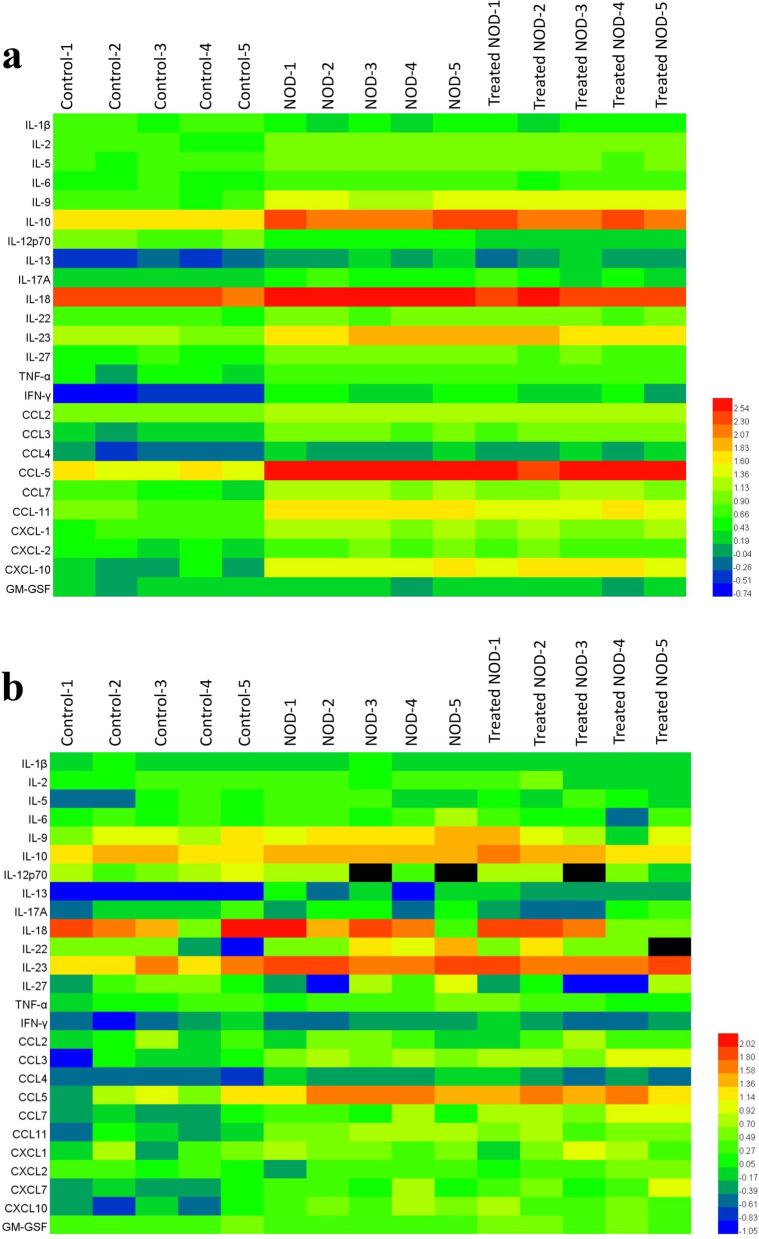
Low dose of IL-2 failed to reduce multiple cytokines or chemokines of the submandibular gland in Sjogren’s syndrome model mice. The heat map of 25 cytokines and chemokines in salivary glands of the SS mice in **a** the short-course group or **b** the long-course group (n = 5)

In SS, a variety of cytokines and chemokines induce immunological damage of the exocrine glands, and the levels of cytokines and chemokines in the exocrine glands also indirectly reflect the immunological damage of the glands. Therefore, we further confirmed the effect of low-dose IL-2 on the submandibular gland immunopathological damage by measuring the levels of cytokines. The results of the short-course experiment showed that the levels of various cytokines and chemokines including IL-1β, IL-2, IL-6, IL-9, IL-10, IL-13, IL-23, IL-12p70, INF-γ, TNF-α, CCL-3, CCL2, CCL4, CCL5, CCL-7, CCL11, CXCL2 and CXCL10 in the submandibular gland of NOD group mice were significantly higher than those in Control mice. The long-course experiment showed that the levels of IL-10, IL-13, IL-23, CCL3, CCL4, CCL5, CCL-7, CCL11, CXCL7 and CXCL10 in the submandibular gland of NOD mice were significantly higher than those in Control mice. However, there was no statistically significant difference in cytokines or chemokines between NOD group and the Treated NOD group, regardless of long-course or short-course treatment, except for the increase in the CCL7 level in the long-term treatment group after IL-2 treatment (Fig. 5a, b).

## Discussion

Treg cells are a subset of CD4^+^ T lymphocytes that highly express IL-2Rα (CD25). They maintain the homeostasis of the immune by mediating immune tolerance to the body’s own factors [27]. One aspect of peripheral self-tolerance is mediated by CD25^+^ T cells, which maintain potentially pathogenic auto-reactive T cells in a CD25 quiescent state. Therefore, in the absence of CD4^+^ T lymphocyte infusion after thymectomy in BALB/c mice, the reduction of autoimmune-preventive CD25^+^ T cells leads to the development of autoimmune gastritis and the emergence of various organ-specific autoimmune diseases [28, 29]. This indicates that Treg cells play an important negative immune regulatory role in preventing or delaying autoimmune diseases. IL-2 has an important role in maintaining the proliferation or activation of Treg cells. Therefore, low doses of IL-2 have been used in the treatment of systemic lupus erythematosus, type 1 diabetes, and other autoimmune diseases. Recent clinical studies have reported that low doses of IL-2 can decrease the levels of hormones and reduce the incidence of infection. Animal experiments have demonstrated that low-doses of IL-2 can enhance the proportion of Treg cells in islet tissues, thereby reducing the incidence of diabetes in mice. It is worth noting that this study did not confirm the finding that low-dose IL-2 can reduce lymphocyte infiltration in pancreatic islet tissues [30]. The results of low-dose IL-2 treatment in an animal model of HLA-B27 transgenic spondylarthritis indicates that, although IL-2 could increase the proportion of Treg cells in spleens, it did not improve the clinical symptom scores and the inflammation of the intestines and joints [31].

Our study demonstrated that a low dose of IL-2 could significantly improve the exocrine function of salivary glands in mice with SS. At the same time, IL-2 up-regulated the proportion of Treg cells in cervical lymph nodes and spleens, which is similar to the results of clinical studies. However, IL-2 did not improve lymphocyte infiltration in the submandibular gland, and it had no significant inhibitory effect on abnormally elevated levels of cytokines or chemokines in the submandibular gland. The above phenomenon seems to overturn a central dogma of biology which states that "structure determines function". However, in SS, the separation between the structure and function of salivary glands is common in clinical practice. Mild lymphocytic infiltration of salivary glands manifests as severe exocrine dysfunction. Although severe salivary lymphocytic infiltration was induced, exocrine gland function was not significantly impaired. The possible explanation may be that the salivary glands may not be the targets of lymphocyte attack. In SS, the alienation of salivary gland cell function may be an inherent property of the disease. For example, the salivary gland epithelial cells of patients with SS can present antigens, release cytokines and chemokines, and express costimulatory molecules, etc. Although low-dose IL-2 did not improve lymphocyte infiltration, it may directly or indirectly improve the function of salivary gland epithelial cells.

CD8^+^ T lymphocytes are considered cytotoxic cells that promote the destruction of the exocrine glands in SS patients [26]. In our experiments, the proportion of CD8^+^ T lymphocytes in the cervical lymph nodes of mice with SS was abnormally increased. In theory, Treg cells can inhibit the killing function of CD8^+^ T lymphocytes, and although IL-2 directly up-regulates the proportion of Treg cells, it had no obvious inhibitory effect on the proportion and function of CD8^+^ T lymphocytes. The possible explanation is that long-term and low-dose IL-2 administration can activate CD8^+^ T lymphocytes, which to some extent counteracts the negative regulatory effect of Treg cells, and this may be due to the abnormally elevated levels of cytokines in exocrine glands. In addition, the unchanged levels of chemokines in the submandibular gland after IL-2 treatment also reasonably explains why it could not improve the infiltration of lymphocytes into the submandibular gland and could not improve the chemotaxis of lymphocytes into the glands, which does not mean that it cannot reverse the decreased salivary gland function.

## Conclusions

Low-dose IL-2 may be a potential treatment for improving salivary gland function in SS patients, but if the disease is further controlled or the structure of the gland is preserved, combination therapy with other immunomodulatory drugs may be a more prudent option.

## Data Availability

The datasets generated and/or analyzed during the current study are not publicly available due to ethical regulation constraints but are available from the corresponding author on reasonable request.

## References

[1] Mavragani CP (2017). Mechanisms and new strategies for primary Sjögren's syndrome. Annu Rev Med.

[2] Baldini C, Talarico R, Tzioufas AG (2012). Classification criteria for Sjogren’s syndrome: a critical review. J Autoimmun.

[3] Alamanos Y, Tsifetaki N, Voulgari PV, Venet-sanopoulou AI, Siozos C, Drosos AA (2006). Epidemiology of primary Sjögren’s syndrome in north-west Greece, 1982–2003. Rheumatology (Oxford).

[4] Mavragani CP, Moutsopoulos HM, The geoepidemiology of Sjögren's syndrome. Autoimm Rev 2010;9(5):A305–A310, ISSN 1568-9972 10.1016/j.autrev.2009.11.004 19903539

[5] Bowman SJ, Ibrahim GH, Holmes G, Ham-burger J, Ainsworth JR (2004). Estimating the prevalence among Caucasian women of primary Sjögren’s syndrome in two general practices in Birmingham. UK Scand J Rheumatol.

[6] Fasano S, Isenberg DA (2019). Present and novel biologic drugs in primary Sjögren's syndrome. Clin Exp Rheumatol.

[7] Helmick CG, Felson DT, Lawrence RC, Gabriel S, Hirsch R, Kwoh CK (2008). Estimates of the prevalence of arthritis and other rheumatic conditions in the United States. Part I. Arthritis Rheum.

[8] Both T, Dalm VA, van Hagen PM, van Daele PL (2017). Reviewing primary Sjögren’s syndrome: beyond the dryness–from pathophysiology to diagnosis and treatment. Int J Med Sci.

[9] Saraux A, Pers JO, Devauchelle-Pensec V (2016). Treatment of primary Sjögren syndrome. Nat Rev Rheumatol.

[10] Ramos-Casals M, Tzioufas AG, Stone JH (2010). Treatment of primary Sjögren syndrome: a systematic review. JAMA.

[11] Price EJ, Rauz S, Tappuni AR (2017). The British Society for Rheumatology guideline for the management of adults with primary Sjögren's Syndrome. Rheumatology.

[12] Dong S, Hiam-Galvez K J, Mowery C T, et al. The effect of low-dose IL-2 and Treg adoptive cell therapy in patients with type 1 diabetes. JCI Insight 2021;6(18) 10.1172/jci.insight.147474 PMC8492314 34324441

[13] He J, Zhang R, Shao M (2020). Efficacy and safety of low-dose IL-2 in the treatment of systemic lupus erythematosus: a randomised, double-blind, placebo-controlled trial. Ann Rheum Dis.

[14] Miao M, Hao Z, Guo Y (2018). Long-term and low-dose il-2 therapy maintains the th17/treg balance in peripheral blood of patients with primary sjÖgren’s syndrome. Ann Rheumatic Dis.

[15] McNally A, Hill GR, Sparwasser T (2011). CD4+ CD25+ regulatory T cells control CD8^+^ T-cell effector differentiation by modulating IL-2 homeostasis. Proc Natl Acad Sci.

[16] Alunno A, Carubbi F, Bistoni O (2014). CD4− CD8− T-cells in primary Sjögren's syndrome: association with the extent of glandular involvement. J Autoimmun.

[17] Rose A, von Spee-Mayer C, Kloke L (2019). IL-2 therapy diminishes renal inflammation and the activity of kidney-infiltrating CD4+ T cells in murine lupus nephritis. Cells.

[18] Dominguez-Villar M, Hafler DA (2018). Regulatory T cells in autoimmune disease. Nat Immunol.

[19] Miao M, Hao Z, Guo Y (2018). Short-term and low-dose IL-2 therapy restores the Th17/Treg balance in the peripheral blood of patients with primary Sjögren’s syndrome. Ann Rheumatic Dis.

[20] Maimela NR, Liu S, Zhang Y (2019). Fates of CD8^+^ T cells in tumor microenvironment. Comput Struct Biotechnol J.

[21] Kim TS, Shin EC (2019). The activation of bystander CD8+ T cells and their roles in viral infection. Exp Mol Med.

[22] Wherry EJ, Ha SJ, Kaech SM (2007). Molecular signature of CD8+ T cell exhaustion during chronic viral infection. Immunity.

[23] Maeno T, Houghton AMG, Quintero PA (2007). CD8+ T Cells are required for inflammation and destruction in cigarette smoke-induced emphysema in mice. J Immunol.

[24] Badovinac VP, Porter BB, Harty JT (2004). CD8+ T cell contraction is controlled by early inflammation. Nat Immunol.

[25] Gravano DM, Hoyer KK (2013). Promotion and prevention of autoimmune disease by CD8+ T cells. J Autoimmun.

[26] Zhou H, Yang J, Tian J (2021). CD8+ T lymphocytes: crucial players in sjögren’s syndrome. Front Immunol.

[27] Sakaguchi S, Sakaguchi N, Asano M, Itoh M, Toda M (1995). Immunologic self-tolerance maintained by activated T cells expressing IL-2 receptor α-chains (CD25):breakdown of a single mechanism of self-tolerance causes various autoimmune diseases. J Immunol.

[28] McHugh RS, Shevach EM (2002). Cutting edge: depletion of CD4+ CD25+ regulatory T cells is necessary, but not sufficient, for induction of organ-specific autoimmune disease. J Immunol.

[29] Asano M, Toda M, Sakaguchi N (1996). Autoimmune disease as a consequence of developmental abnormality of a T cell subpopulation. J Exp Med.

[30] Grinberg-Bleyer Y, Baeyens A, You S (2010). IL-2 reverses established type 1 diabetes in NOD mice by a local effect on pancreatic regulatory T cells. J Exp Med.

[31] Araujo LM, Jouhault Q, Fert I (2021). Effects of a low-dose IL-2 treatment in HLA-B27 transgenic rat model of spondyloarthritis. Arthritis Res Ther.

